# Single-shot lensless imaging with fresnel zone aperture and incoherent illumination

**DOI:** 10.1038/s41377-020-0289-9

**Published:** 2020-04-07

**Authors:** Jiachen Wu, Hua Zhang, Wenhui Zhang, Guofan Jin, Liangcai Cao, George Barbastathis

**Affiliations:** 1 State Key Laboratory of Precision Measurement Technology and Instruments, Department of Precision Instruments, Tsinghua University, 100084 Beijing, China; 2 Department of Mechanical Engineering, Massachusetts Institute of Technology, 77 Massachusetts Avenue, Cambridge, MA 02139 USA

**Keywords:** Optoelectronic devices and components, Imaging and sensing

## Abstract

Lensless imaging eliminates the need for geometric isomorphism between a scene and an image while allowing the construction of compact, lightweight imaging systems. However, a challenging inverse problem remains due to the low reconstructed signal-to-noise ratio. Current implementations require multiple masks or multiple shots to denoise the reconstruction. We propose single-shot lensless imaging with a Fresnel zone aperture and incoherent illumination. By using the Fresnel zone aperture to encode the incoherent rays in wavefront-like form, the captured pattern has the same form as the inline hologram. Since conventional backpropagation reconstruction is troubled by the twin-image problem, we show that the compressive sensing algorithm is effective in removing this twin-image artifact due to the sparsity in natural scenes. The reconstruction with a significantly improved signal-to-noise ratio from a single-shot image promotes a camera architecture that is flat and reliable in its structure and free of the need for strict calibration.

## Lensless imaging: reduced noise

Two simple innovations have been found to significantly improve the quality of lensless imaging. Jiachen Wu and coworkers from Tsinghua University in China and MIT in the US report that introducing a Fresnel optical element and applying a compressive sensing algorithm can significantly reduce the level of noise in reconstructed images. Their simple lensless system consists of a Fresnel Zone Aperture (FZA) placed 3 mm in front of a CMOS image sensor. The FZA is a mask of patterned chrome that consists of a series of concentric rings that alternatingly transmit and block light. Images on an LCD screen placed ~30 cm from the mask are used as a test object. The signal recorded by the CMOS sensor is then reconstructed by a compressive sensing algorithm with total variation denoising to generate an improved image of the object.

## Introduction

Traditional optical imaging architectures follow a point-to-point imaging model by using a set of lenses. This way to collect and converge light limits the freedom of imaging parameters. By introducing computing capability into the imaging system, breakthrough systems have been built, yielding improvements in the image dimensions^1–3^, image size^4,5^, and even imaging mechanisms^6–8^. Furthermore, the burden of imaging can be transferred from bulky and expensive hardware to computation, which enables new architectures for low-cost cameras.

Recently, lensless imaging has become attractive due to its thin and easy-to-build form. In the past few years, various lensless imaging techniques have been proposed with coherent systems, such as an on-chip microscope^9,10^, coherent diffractive imaging^11,12^, and a series of learning-based methods^13–15^. Due to the requirement of coherent illumination, however, applications of such systems are limited. The pinhole camera offers a simple architecture for incoherent light imaging, which has an infinite depth of field and no aberration but suffers from the severe limitation of low light throughput. To make up for this drawback, coded aperture imaging replaces the pinhole with a mask. Each light source in the scene casts a unique shadow of the mask onto the sensor, encoding the intensity and the location of the light source. Masks, which include a uniform redundant array^16^ or modified URA^17^, have traditionally been used in X-ray and γ-ray imaging. Several lensless cameras at visible wavelengths have been proposed using diffractive gratings that are insensitive to wavelength^18,19^, separable masks^20,21^ and diffusers^22^. These methods depend on large-scale matrix inversion, which requires strict calibration and heavy computational resources.

Unlike the pure mathematical optimization methods mentioned above, we adopt the Fresnel zone plate as the mask to improve the condition of the imaging problem at the physical level. Soon after Gabor’s invention of holography^23^, Rogers noted that the pattern of the Fresnel zone plate coincides with a point source hologram^24^. Inspired by this, Mertz and Young proposed zone plate coded imaging^25^ and extended the concept and application of holography to the field of incoherent light. Recent works have demonstrated the imaging capability of the Fresnel zone plate, also called the Fresnel zone aperture (FZA), at visible wavelengths^26–29^. This prototype camera needs at least four FZAs with different phases to extract the signal from captured images. Time division and spatial division are two alternatives to implement the change of masks. The former increases the complexity of the system by using a spatial light modulator. The latter reduces the resolution because the size of the reconstruction image is only a quarter of the sensor size. Moreover, the crosstalk of light and sensor noise would further degrade the quality of the reconstructed image.

In mask-based imaging systems, image reconstruction is susceptible to noise, so a robust reconstruction algorithm is critical. Compressive sensing (CS) is a powerful signal reconstruction framework and provides complete theoretical support for image reconstruction^30^. The CS algorithm has been widely applied in optical imaging systems, such as single pixel cameras^31–33^ and compressive holography^34–38^. These previous works pave the way for inverse problem solving in optical imaging and inspire us in terms of image reconstruction.

**Fig. 1:**
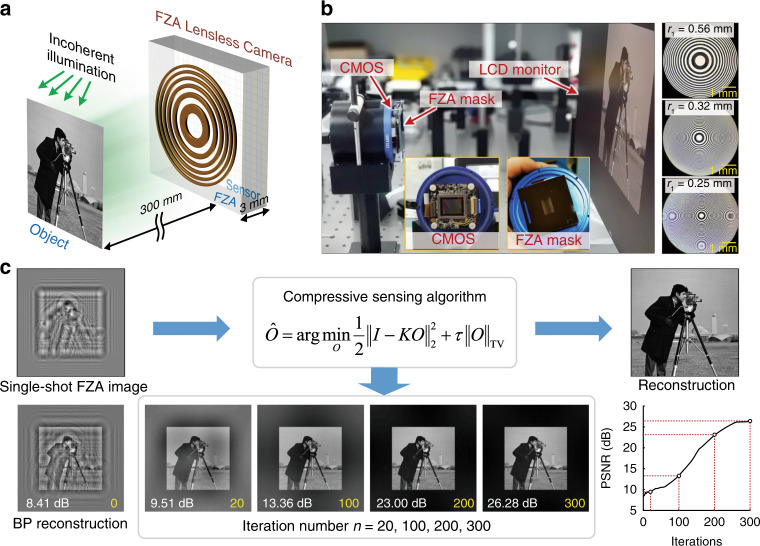
Overview of FZA lensless imaging. **a** Sketch of the apparatus. An object is placed at a distance of *z1* = 300 mm from an FZA mask and is illuminated by incoherent light. The FZA mask is placed at a distance of *z2* = 3 mm from the image sensor. **b** Experimental apparatus. The FZA mask is placed close to the CMOS sensor. Three FZA masks with different *r1* values were tested in the experiment. The size of each pattern is 20 mm × 20 mm. **c** The image of the object is reconstructed from the single-shot FZA image by using a compressive sensing algorithm. The BP reconstruction is employed as the initial value for the CS algorithm. The reconstructions are presented after 20, 100, 200, 300 iterations, which are marked in the peak signal to noise ratio (PSNR) curve

In this work, we propose a single-shot noise suppression lensless imaging method with FZA and incoherent illumination. The structure of the proposed imaging system is simple: an FZA mask is placed close to a sensor (Fig. 1a) without precise alignment. Each point source in the scene casts an FZA shadow on the sensor plane, which is similar to the point source hologram, so that the FZA acts in some sense as a Fresnel hologram encoder. Backpropagation (BP) reconstruction is feasible but disturbed by twin image. Here, the CS algorithm that has been proven to be efficient for Fresnel holography is applied (Fig. 1c). By enforcing a sparsity constraint in the gradient domain, the reconstruction eliminates the twin-image effect and suppresses the other noise. Since the single-shot method only needs a single FZA, all the pixels can be saved for a full-size reconstruction image, while in the multiple-shot method, only a quarter size can be realized. The proposed method can greatly support high-quality imaging without the need for strict calibration for the FZA lensless camera.

## Results

A lensless camera using a QHY163M CMOS image sensor is designed and arranged as shown in Fig. 1b. The size of the image sensor is 17.7 mm × 13.4 mm, and the number of pixels is 4656 × 3522. The pixel pitch is 3.8 μm. The captured image is cropped to 2048 × 2048 pixels for subsequent processing. An FZA mask is placed close to the CMOS sensor. The transmission function of the FZA mask is

1 $$T\left( r \right)\,=\,\frac{1}{2}\,+\,\frac{1}{2}{\rm{sgn}} \left[ \cos \left( {\frac{{\pi r^2}}{{r_1^2}}} \right) \right]$$

where *r*_1_ denotes the radius of the innermost zone and *r* is the radial distance from the center of the aperture. In addition, “sgn” is the signum function that is +1 for a positive number or −1 for a negative number. We use three FZAs with different *r*_1_ to test the imaging resolution. The thickness of the photomask is 2 mm, and the thickness of the protective glass is 1 mm. Thus, the distance between the mask and the sensor is 3 mm. An LCD monitor with 1920 × 1080 resolution is placed ~300 mm from the FZA. The test images are displayed on the screen, and the sensor records the rays from the screen that are modulated by the mask. For each test image, we adjusted the exposure time according to the scene intensity to avoid overexposure or underexposure.

**Fig. 2:**
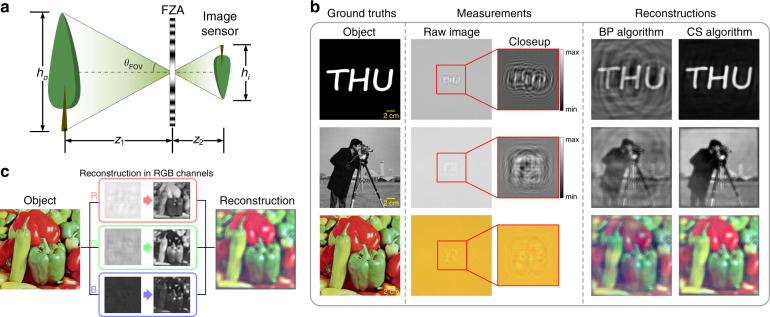
Experimental results using the FZA lensless camera. **a** The FOV of the FZA lensless camera. **b** The reconstructions for the binary, grayscale and color images. **c** The process of color imaging is reconstructing RGB channels independently and combining them into color

The FZA imaging follows the pinhole imaging model. The position of each reconstructed image point on the sensor plane is at the intersection of the chief ray passing through the center of the FZA and the sensor plane (Fig. 2a). The magnification of the system is derived from the geometrical relationship:

2 $$M\,=\,\frac{{h_i}}{{h_o}}\,=\,\frac{{z_2}}{{z_1}}$$

where *h*_*o*_ is the object height and *h*_*i*_ is the image height. The field of view (FOV) is expressed as

3 $$\theta _{{\mathrm{FOV}}}\,=\,\arctan \left( {\frac{{h_i}}{{2z_2}}} \right)$$

The binary, grayscale and color images are tested in our experiments. The original images, the corresponding measured images, and the reconstruction results are shown in Fig. 2b. The original image is 20 cm × 20 cm when displayed on screen. The measured images are monochrome images with 16-bit depth. For binary and grayscale images, the closeups of the raw images normalize the grayscale of the measured images. The BP and CS algorithms are used to reconstruct images from measured images. According to the magnification, the central 526 × 526 portions of each image are cropped to be shown in Fig. 2b.

The FZA lensless camera has the capacity for color imaging as well. Because the imaging model is based on geometrical optics, the reconstruction is not influenced by the wavelength of the object. By using an RGB sensor, the intensity of different wavelength bands can be recorded by the RGB channels independently and the reconstruction algorithm applied to the three channels. Then, the reconstructed images of the three channels are combined into the final color image (Fig. 2c).

## Discussion

### Resolution

In practice, the sensor and the FZA mask both have finite sizes, which means that the reconstruction is band-limited. If the sensor can completely record the FZA shadow and the pixel pitch is small enough to satisfy the sampling theorem, the frequency range is limited by the number of recorded zones, which in turn depends on the radius of the FZA. The larger the radius, the finer the zones that are included. Thus, the resolution of the reconstructed image can be improved by increasing the aperture radius *R*. On the other hand, the FZA constant *r*_1_ also determines the number of zones within a fixed aperture radius *R*. Improving the resolution of the reconstructed image can also be realized by shrinking the FZA constant *r*_1_.

Quantitative analysis is carried out by means of the coherent impulse response (CIR). As shown in section “Resolution”, the CIR of the imaging system is calculated as

4 $$I_{{\mathrm{CIR}}}\left( {r_o} \right)\,=\,\exp \left( {\frac{{i\pi }}{{r_1^2}}r_0^2} \right)\frac{R}{{r_0}}J_1\left( {2\pi r_0R/r_1^2} \right)$$

where *R* is the radius of FZA and *J*_1_(·) is the first-order Bessel function of the first kind. Because the intensity of the object is real-valued, only the real part of the CIR should be considered. According to the Rayleigh criterion, the minimum distance between resolvable points is defined as the distance from the center to the first zero of the CIR. The first zero of the real part of the exponential term is 0.707*r*_1_, whereas the first zero of the order-one Bessel function is $0.61\left( {r_1/R} \right)r_1$. Since *r*_1_ << *R*, the resolution is determined by

5 $$r_c\,=\,0.61\frac{{r_1^2}}{R}$$

Assuming the FZA contains *n* zones, namely, $R\,=\,\sqrt n r_1$, then the width of the outermost zone is $\Delta r\,=\,\left( {\sqrt n\,-\,\sqrt {n\,-\,1} } \right)r_1\,\approx\, r_1/\left( {2\sqrt n } \right)$. In terms of Δ*r*, the resolution is expressed as

6 $$r_c\,=\,1.22\Delta r$$

Equation (6) reveals the simple yet useful result that the resolution of an FZA imaging system is approximately equal to the width of the outermost zone. Taking the system magnification *M* into account, the resolution at the object plane is $r_{c}^{\prime}\,=\,\left( {1/M} \right)r_c$.

**Fig. 3:**
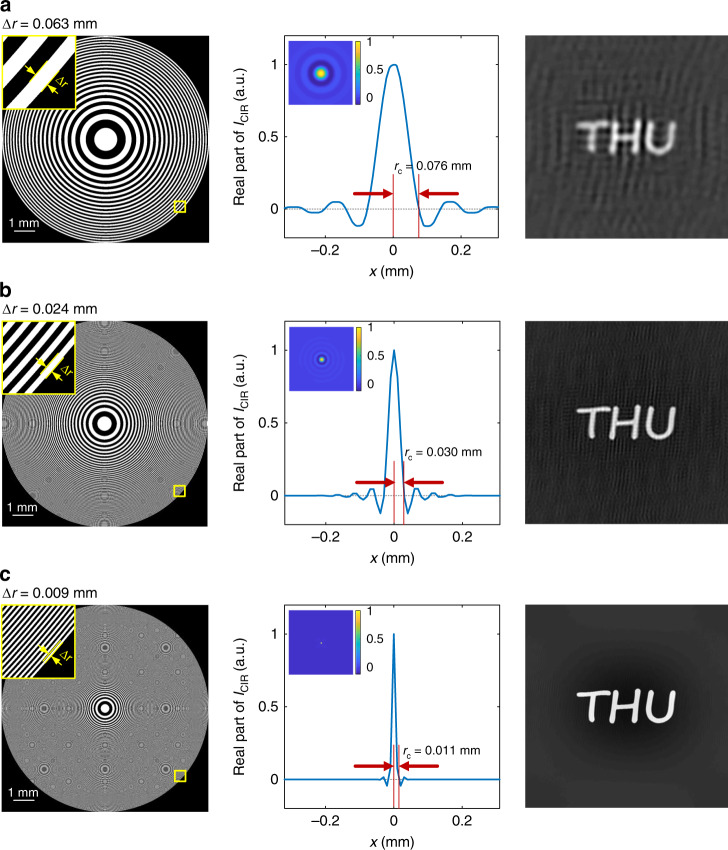
Image resolution contrast of the FZA imaging system with different FZA constants *r1*. The left to right columns show the FZAs (the outermost zones are shown in the top left insets), the CIRs (the 2D distributions are shown in the top left insets), and the reconstructed images. The values of *r1* from top to bottom in rows are **a** 0.8 mm, **b** 0.5 mm, and **c** 0.3 mm

Figure 3 shows the CIRs and the corresponding reconstructed images with different values of *r*_1_. The aperture radius *R* = 5.12 mm, and the values of *r*_1_ are 0.8, 0.5, and 0.3 mm. The corresponding values of *r*_c_ and Δ*r* are labeled in Fig. 3, which agrees with Eq. (6). The smaller FZA constant *r*_1_ yields higher-quality reconstructions, which verifies the above discussion. Note that the resolution improvement is limited because when the FZA constant decreases, diffraction becomes noticeable, which obfuscates the reconstruction.

**Fig. 4:**
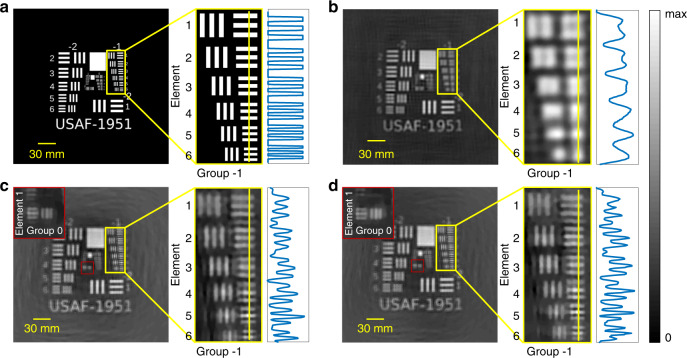
Experimental test of the spatial resolution. Group-1 with intensity profiles is shown on the right side of each figure for comparison. The ground truth shown in **a** is a negative USAF 1951 test chart that is uploaded on the monitor with three times magnification. The results are reconstructed by using the FZA with **b** *r1* = 0.56 mm, **c** *r1* = 0.32 mm, and **d** *r1* = 0.25 mm. The insets on top left of **c** and **d** show the magnification results of group 0/element 1, which are barely resolved

A negative USAF 1951 test chart is displayed on the monitor to experimentally test the resolution. The displayed size has a magnification three times larger than the standard size. Three FZAs with the same aperture radius *R* and different FZA constants *r*_1_ are used for comparison. The FZA constants *r*_1_ are 0.56, 0.32, and 0.25 mm. The aperture size is approximately equal to the sum of the whole image size and object imaging size, and the aperture radius is 4.56 mm. The results are shown in Fig. 4. For *r*_1_ = 0.56 mm, group-2/element 5 can barely be resolved (Fig. 4b), which represents that the minimum resolved distance is 3.78 mm at the object plane (or 0.038 mm at the image plane, after multiplying by the magnification 0.01). For *r*_1_ = 0.32 mm, group 0/element 1 can barely be resolved (Fig. 4c), and the corresponding minimum resolved distance is 1.5 mm in the object plane and 0.015 mm in the image plane. The experimental values are close to the theoretical values, which are 0.042 mm and 0.014 mm calculated by Eq. (5), respectively. When *r*_1_ reduces to 0.25 mm, the resolution does not improve significantly (Fig. 4d); that is, the diffraction effect limits the resolution.

### Noise and error analysis

Except for the twin-image noise, the measurement can be corrupted by other noises and errors originating mainly from three sources: sensor, mask, and diffraction. The sensor noise consists of quantization noise and dark current noise. The effect of this noise on the reconstruction is relatively moderate and can be reduced by improving bit depth and increasing exposure time.

The mask error is caused by binarization of transmittance. The binary FZA, as a substitute of the GZP, consists of only transparent and opaque zones. However, in comparison to the GZP, which has only one pair of conjugate foci at $f\,=\,\pm r_1^2/\lambda$, the FZA has multiple foci with corresponding focal lengths $f\,=\,\pm r_1^2/n\lambda ,\,n\,=\,1,3,5...$. The property can be explained by using Fourier expansion. The Fourier series representation of this binary function is

7 $$T\left( r \right)\,=\,\frac{1}{2}\,+\,\frac{2}{\pi }\mathop {\sum}\limits_{n\,=\,1}^\infty {\frac{1}{n}\sin \left( {\frac{{n\pi }}{2}} \right)\cos \left( {\frac{{\pi nr^2}}{{r_1^2}}} \right)}$$

The derivation can be seen in the supplementary information (Section S1). Equation (7) states that the intensity transmittance function of the FZA is a line0ar superposition of a series of GZPs with different focal lengths. The higher-order focal lengths cause a series of defocused images at the first-order focal plane, which degrades the image quality. This issue can be remedied by using a binary Gabor zone plate (GZP) that has only one pair of conjugate foci^39,40^. Increasing the regularization coefficient appropriately can also effectively suppress these defocused noises.

**Fig. 5:**
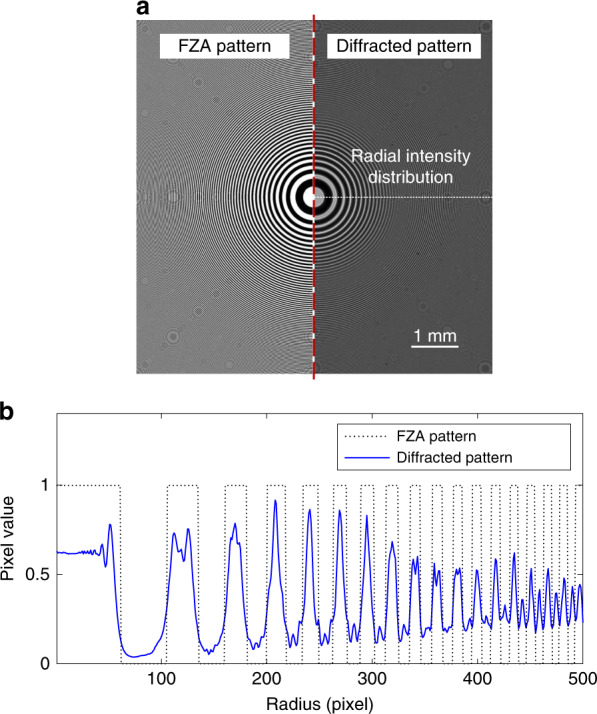
Experimental evaluation of the difference between the FZA pattern and diffracted pattern. **a** Comparison of the FZA pattern and the diffracted pattern. **b** Radial intensity distribution of the diffracted FZA pattern along the axis inspected in **a**

With a decreasing zone width, diffraction effects become noticeable. The diffracted pattern is no longer an ideal FZA pattern. To observe the diffraction phenomena, a white collimating LED light is adopted to illuminate the FZA mask. The FZA pattern with *r*_1_ = 0.32 mm and the corresponding diffracted pattern are shown in Fig. 5a. The cross section of the radial intensity is shown in Fig. 5b. The contrast drops to the lowest value around a radius of 500 pixels away from the center due to the diffraction propagation law of objects with periodic transmittance. Since the contrast of the diffraction fringe of grating changes periodically along with propagation distance, the contrast is minimized when the propagation distance is an odd number of half Talbot lengths^41^; that is,

8 $$z^{(\min )}\,=\,l\,+\,\frac{1}{2}z_T\,=\,l\,+\,\frac{{p^2}}{{2\lambda }},\;l\,=\,0,1,2, \ldots$$

where *p* is the grating period. The distance is fixed, but the period $p\,=\,r_1^2/r$ varies with the radius. Thus, the contrast also varies with the radius. This decrease in contrast results in model error and limits the reconstruction resolution. The mask can be improved by adopting a well-designed diffraction optical element to obtain the desired diffraction pattern in future work.

### Outlook

We have proposed an FZA lensless imaging method under incoherent illumination using computational reconstruction. CS theory provides a guarantee for accurate signal reconstruction. The proposed camera consists of an image sensor and an FZA mask and thus is thin and compact. The incoherent rays pass through the FZA and cast object-dependent shadows on the sensor plane to compose the raw image. This mechanism is somewhat analogous to that of inline holography. Unlike conventional BP, which produces twin images, the compressive sensing algorithm with total variation (TV) regularization that we adopt here eliminates the twin-image effect and reconstructs the image with reduced noise.

The imaging resolution of this mask-based camera still has much room for further improvements by modifying the mask so that the diffracted pattern coincides with the expected pattern. The prospect of FZA imaging using such a relatively thin setup can lead to all kinds of applications. One is a portable camera to image various objects from a person in a room to the landscape outdoors. This approach can fulfill a range of recognition tasks, such as object detection, character recognition, and face recognition. Furthermore, the proposed camera can be monolithically fabricated by depositing the FZP pattern on the cover glass of the sensor so that it can be readily integrated with portable devices or any flat surface. It is suggested that this ultrathin and low-cost camera has great potential with the development of computational imaging.

## Materials and methods

### Imaging model

An ideal zone plate is called a GZP. Its amplitude transmission function is

9 $$T\left( r \right)\,=\,\frac{1}{2}\,+\,\frac{1}{2}\cos \left( {\frac{{\pi r^2}}{{r_1^2}}} \right)$$

**Fig. 6:**
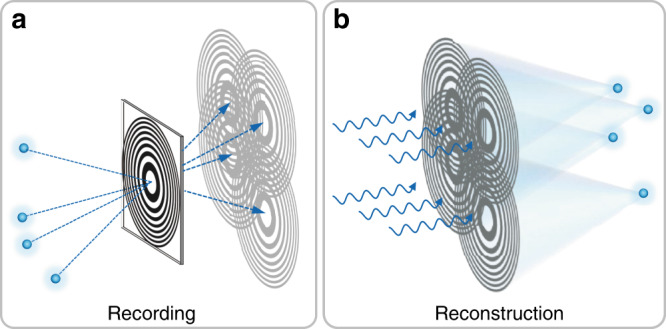
Recording and reconstruction of FZA imaging. **a** Each point source in the scene casts an FZA shadow on the sensor plane. These shadows are superimposed and form the pattern with the same form as the inline hologram. **b** Applying coherent propagation for the recorded pattern can reconstruct the point sources

However, such a zone plate is difficult to manufacture because of its sinusoidal variation transmittance. The FZA with binary transmission is a more practical alternative mask. For the sake of derivation, we substitute the transmission function of the GZP for the FZA in subsequent content. The object is placed at a distance of *z*_1_ from the FZA and illuminated by an incoherent light source. The FZA is placed in front of an image sensor at distance *z*_2_. The object surface diffuses the light and can be considered a superposition of point sources. Each point source casts an FZA shadow on the sensor plane (Fig. 6a). The shadow center is at the intersection of the chief ray and the sensor plane. The size of the shadow is expanded from the FZA by the magnification factor (1 + *z*_2_/*z*_1_). Then, the FZA constant of the shadow would be $r_{1}^{\prime} = \left( {1 + z_2/z_1} \right)r_1$. Thus, the image formed on the sensor is a superposition of shifted and scaled versions of FZA shadows. When *z*_1_ >> *z*_2_, the FZA constant *r*_1_′ is almost equal to *r*_1_. In this case, the imaging process can be formulated as

10 $$I\left( {\mathbf{r}} \right)\,=\,\frac{1}{2}\mathop {\sum}\limits_k^N {I_k} \left[ {1 + \cos \left( {\frac{\pi }{{r_1^2}}\left| {{\mathbf{r}}\,-\,{\mathbf{r}}_k} \right|^2} \right)} \right]$$

where *I*(**r**) is the intensity distribution on the sensor plane; *I*_*k*_ is the intensity of the *k*th point source; the vector **r** is the arbitrary position vector, and **r**_*k*_ is the translation vector of the *k*th shadow in the sensor plane. Each FZA shadow can be considered a point source hologram that encodes the intensity and the location of the point source. All these elementary holograms synthesize the final measurement. Then, the reconstruction can be performed by coherent propagation (Fig. 6b). Both optical and computational methods are available for the reconstruction.

In the Fresnel approximation, the reconstructed wavefront may be written as

11 $$O_R\left( {{\mathbf{r}}_o} \right)\,=\,\frac{{\exp \left( {i2\pi d/\lambda } \right)}}{{i\lambda d}}{\int\!\!\!\!\!\int} {I\left( {\mathbf{r}} \right)\exp \left[ {\frac{{i\pi }}{{\lambda d}}\left| {{\mathbf{r}}\,-\,{\mathbf{r}}_o} \right|^2} \right]dS}$$

where *λ* and *d* represent the wavelength of the reconstructed wavefront and the reconstructed distance, respectively. To ensure correct reconstruction, the two parameters should satisfy $r_1^2 = \lambda d$. The vector **r**_*o*_ is the position vector in the reconstructed plane. Here, *dS* is the area element in the measured image. For the ideal case, the integral area is infinite. Expanding the cosine term in Eq. (10) into an exponential form and substituting it into Eq. (11) while ignoring the constant coefficient, Eq. (11) becomes

12 $$\begin{array}{l}O_R\left( {{\mathbf{r}}_o} \right)\,=\,\frac{1}{2}{\int\!\!\!\int} {\exp \left( {\frac{{i\pi }}{{r_1^2}}\left| {{\mathbf{r}} - {\mathbf{r}}_o} \right|^2} \right)dS} \cdot \mathop {\sum}\limits_k^N {I_k} \\\qquad\qquad\quad+\,\frac{1}{4}\mathop {\sum}\limits_k^N {I_k} {\int\!\!\!\int} {\exp \left[ {\frac{{i\pi }}{{r_1^2}}\left( {\left| {{\mathbf{r}}\,-\,{\mathbf{r}}_o} \right|^2\,-\,\left| {{\mathbf{r}}\,-\,{\mathbf{r}}_k} \right|^2} \right)} \right]dS} \\\qquad\qquad\quad + \frac{1}{4}\mathop {\sum}\limits_k^N {I_k} {\int\!\!\!\int} {\exp \left[ {\frac{{i\pi }}{{r_1^2}}\left( {\left| {{\mathbf{r}}\,-\,{\mathbf{r}}_o} \right|^2\,+\,\left| {{\mathbf{r}}\,-\,{\mathbf{r}}_k} \right|^2} \right)} \right]dS} \\\qquad\quad = \frac{{ir_1^2}}{2}\mathop {\sum}\limits_k^N {I_k} + \frac{{r_1^4}}{4}\mathop {\sum}\limits_k^N {I_k\delta \left( {{\mathbf{r}}_o\,-\,{\mathbf{r}}_k} \right)} \\\qquad\qquad\quad + \frac{{ir_1^2}}{8}\mathop {\sum}\limits_k^N {I_k\exp \left( {\frac{{i\pi }}{{2r_1^2}}\left| {{\mathbf{r}}_o\,-\,{\mathbf{r}}_k} \right|^2} \right)} \end{array}$$

The first term is a constant term that is proportional to the total intensity of the object. The second term is a set of points appearing at the same locations as the geometrical imaging point, and the intensities are proportional to the intensities of the original light sources. These points reproduce the image of the original object. The third term is the superposition of spherical waves propagating from distance 2*d*. It can be considered an out-of-focus image, which is the so-called twin image. The inherent twin image obscures the reconstruction. The most common methods to remove twin images by experimental means, such as off-axis holography^42^ and phase-shifting^43^, are not applicable in this case. Instead, we use a computational guarantee in the form of compressive sensing toward a twin-image-free reconstruction.

For the finite size in the real system, the reconstructed image point is no longer a delta function. By introducing the aperture function $A\left( r \right)\,=\,{\mathrm{circ}}\left( {r/R} \right)$ into the integral, the CIR of the imaging system after eliminating the twin image is calculated by setting **r**_*k*_ = 0 and *I*_*k*_ = 1 in the second term of Eq. (12), which is

13 $$\begin{array}{c}I_{{\mathrm{PSF}}}\left( {r_o} \right)\,=\,{\int\!\!\!\int} {\exp \left[ {\frac{{i\pi }}{{r_1^2}}\left( {\left| {{\mathbf{r}}\,-\,{\mathbf{r}}_o} \right|^2\,-\,\left| {\mathbf{r}} \right|^2} \right)} \right]A\left( {\left| {\mathbf{r}} \right|} \right)dS} \\ = \exp \left( {\frac{{i\pi }}{{r_1^2}}r_0^2} \right)\frac{R}{{r_0}}J_1\left( {2\pi r_0R/r_1^2} \right)\end{array}$$

### Reconstruction algorithm

The captured image may be represented as the convolution of the ideal image and FZA shadow by rewriting Eq. (10) as

14 $$I\left( {x,y} \right)\,=\,O\left( {x,y} \right)\,\ast\,T\left( {x,y} \right)\,+\,e\left( {x,y} \right)$$

where “*” denotes the convolution. *O*(*x*, *y*) is the image to be restored on the sensor plane. *e*(*x*, *y*) is a random term that includes photodetector noise, crosstalk, quantization noise and artifacts caused by diffraction. If we divide the cosine term of *T*(*x*, *y*) into $\left[ {h\left( {x,y} \right)\,+\,h^ \ast \left( {x,y} \right)} \right]/2$, where $h\left( {x,y} \right)=\exp \left[ {i\left( {\pi /r_1^2} \right)\left( {x^2\,+\,y^2} \right)} \right]$ and h* is the conjugate of *h* to express *T*(*x*, *y*), Eq. (14) becomes

15 $$\begin{array}{lll}I\left( {x,y} \right)\,&=&\,C\,+\,\frac{1}{4}\left[ {O\left( {x,y} \right)\,\times\,h\left( {x,y} \right)} \right.\\ &&\left. { + O\left( {x,y} \right)\,\ast\,h^ \ast \left( {x,y} \right)} \right]\,+\,e\left( {x,y} \right)\\ &=& C + \frac{1}{4}U\left( {x,y} \right)\,+\,\frac{1}{4}U^ \ast \left( {x,y} \right) + e\left( {x,y} \right)\\ &=& C + \frac{1}{2}{\rm{Re}} \left\{ {U\left( {x,y} \right)} \right\}\,+\,e\left( {x,y} \right)\end{array}$$

where $C\,=\,O\left( {x,y} \right)\, \times \,(1/2)$ is a constant. Here, *h*(*x*, *y*) has the same form as the Fresnel propagation kernel $\exp \left[ {i\left( {\pi /\lambda d} \right)\left( {x^2\,+\,y^2} \right)} \right]$ when $r_1^2\,=\,\lambda d$. $U\left( {x,y} \right)$ can be regarded as the diffracted wavefront of propagating at the virtual distance of *d* and the virtual wavelength of *λ*. $U^ \ast \left( {x,y} \right)$ is the conjugate wave of $U\left( {x,y} \right)$. Equation (15) indicates that the measurement has the same form as the inline hologram except for the background intensity $\left| U \right|^2$.

Let us denote by $N_x\,\times\,N_y\,=\,N_{\mathrm{xy}}$ the number of im *O*(*x*, *y*) age samples. The constant term in Eq. (15) can be removed by filtering out the direct current component. Then, the measured image $I \in {\Bbb R}^{N_{xy}}$ is expressed as a function related to $O \in {\Bbb R}^{N_{xy}}$, which is the forward transform model:

16 $$I\,=\,\frac{1}{2}{\rm{Re}} \left\{ {{\mathcal{F}}^{ - 1}{\mathcal{HF}}{\it{O}}} \right\}\,+\,e$$

where ${\mathcal{F}}$ and ${\mathcal{F}}$^−1^ are the Fourier transform operator and inverse Fourier transform operator, respectively, and ${\mathcal{H}}$ indicates the operator that multiplies by the transfer function $H = i\exp \left[ { - i\pi \lambda z\left( {u^2 + v^2} \right)} \right]$. Since *O* is a real function and *H* is a central symmetry function, Eq. (16) can be written as

17 $$I\,=\,\frac{1}{2}{\mathcal{F}}^{-1}{\mathcal{H}}_T{\mathcal{F}}{O\,+\,e}$$

where ${\mathcal{H}}$_*T*_ indicates the operator multiplied by $H_T\,=\,{\rm{Re}} \left\{ H \right\}\,=\,\sin \left[ {\pi r_1^2\left( {u^2\,+\,v^2} \right)} \right]$, which is the normalized Fourier transform of $\cos \left[ {\pi \left( {x^2\,+\,y^2} \right)/r_1^2} \right]$. Equation (17) is the representation of Eq. (14) in the frequency domain. Let $F \in {\Bbb C}^{N_{xy}\,\times\,N_{xy}}$ be a 2D discrete Fourier transform matrix. Denote Σ as a diagonal matrix whose nonzero entries are the discrete value of *H*_*T*_. Then, the observation *I* is given by *I* = *KO*, where *K* is

18 $$K\,=\,F{^\ast}\Sigma F$$

Solving *O* with a known *I* and forward transform *K* is a typical inverse problem. The solution is not unique because any value can be freely assigned to the imaginary part of *U*(*x*, *y*) in Eq. (15). To remedy this issue, a priori knowledge as a regularization item should be integrated into the process of image reconstruction to obtain a stable solution. For natural images, the gradient distribution tends to be zero, and object *O* can be regarded as sparse in the gradient domain. In contrast, the twin image generates a diffuse pattern, which is nonsparse in the gradient domain. Thus, the sparsity constraint can eliminate the twin image.

Therefore, the reconstruction can be realized by minimizing the objective function:

19 $$\hat O\,=\,\arg \mathop{\rm{min}}\limits_{O}\frac{1}{2}\left\| {I\,-\,{\mathrm{KO}}} \right\|_2^2\,+\,\tau {\mathrm{\Phi }}$$

where Φ is the regularizer imposing the sparsity constraint and ||·||_2_ denotes the $\ell _2$ norm. The regularization parameter *τ* controls the relative weight of the two terms. To guarantee accurate reconstruction, the observation matrix should be subject to a restricted isometry property (RIP) condition according to compressive sensing theory^44^. Note that *K* is a block-circulant matrix, which is under the RIP condition with very high probability^45^. With a suitable regularizer, the objective function can converge rapidly and acquire a good result. Since *K*is related to the reconstruction distance, the method can realize numerical focusing for 3D scenes. The axial resolution is analyzed in the supplementary section (Section S2).

### Total variation denoising

Exploiting the sparsity of the unknown image can significantly enhance reconstruction performance. The $\ell _1$ norm and TV are widely used regularization methods. For natural scenes, TV regularization usually works better than $\ell _1$ regularization. The TV of an image is given by the sum of magnitudes of the image gradients as

20 $$\left\| O \right\|_{{\mathrm{TV}}}\,=\,\mathop {\sum}\limits_{i}^{N_{xy}}\sqrt {|\Delta _i^hO|^2 + |\Delta _i^vO|^2}$$

where $\Delta _i^h$ and $\Delta _i^v$ denote the horizontal and vertical first-order local difference operations, respectively. Since the in-focus object has sharp edges while the out-of-focus twin image is diffuse, the TV of the in-focus image is much less than that of the twin image. The reconstruction can be represented in the form of the following TV minimization problem:

21 $$\hat O\,=\,\arg \mathop{\rm{min }}\limits_{O} \frac{1}{2}\left\| {I\,-\,{KO}} \right\|_2^2\,+\,\tau \left\| O \right\|_{{\mathrm{TV}}}$$

**Fig. 7:**
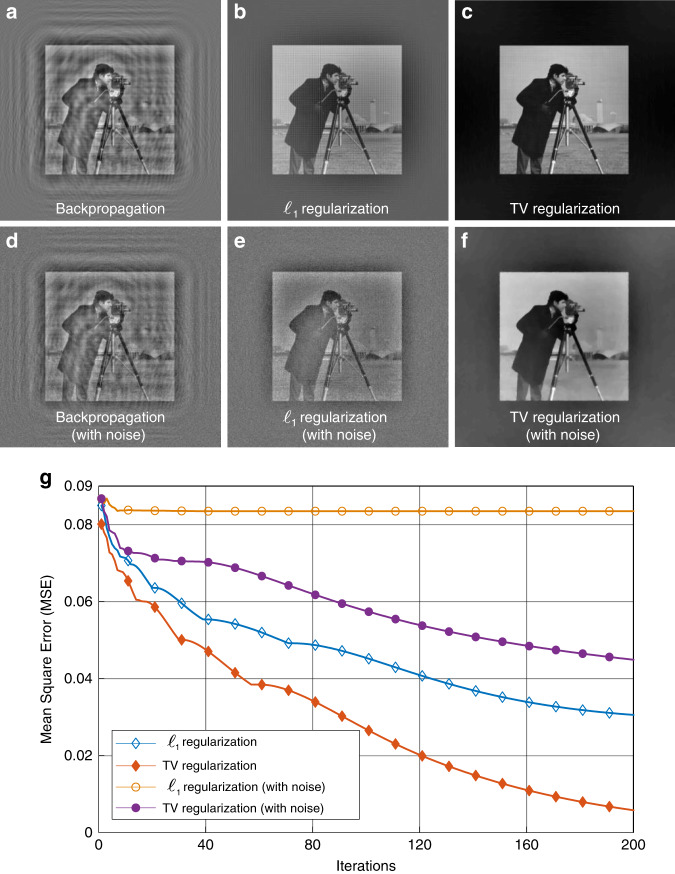
Image reconstruction simulation with and without noise for a grayscale image. **a** Backpropagation reconstruction. **b**, **c** Reconstruction using and TV regularization from a noise-free observation after 200 iterations. **d** Backpropagation reconstruction from a Gaussian-noised image. **e**, **f** Reconstruction using and TV regularization from a Gaussian-noised image after 200 iterations. **g** MSE vs. iteration order. See also Movie 1

We adapt the two-step iterative shrinkage/thresholding (TwIST) algorithm^46^ to solve Eq. (21). The TwIST algorithm is a nonlinear two-step iterative version of the iterative shrinkage/thresholding (IST) algorithm to improve the convergence rate. The TwIST algorithm provides $\ell _1$ regularization by default. It can also be extended to TV regularization by passing a function handle. The corresponding code can be found in GitHub^47^. The performance of TV regularization and $\ell _1$ regularization is shown in Fig. 7. In this case, *z*_1_ = 200 mm, *z*_2_ = 3 mm, and the size of the object is 200 mm × 200 mm. The mean square error (MSE) is introduced to evaluate reconstruction quality quantitatively. The MSE of the reconstruction error is defined as

22 $${\mathrm{MSE}}\,=\,\frac{1}{{N_{\mathrm{xy}}}}\mathop {\sum}\limits_{i\,=\,1}^{N_x} {\mathop {\sum}\limits_{j\,=\,1}^{N_y} {\left[ {O\left( {i,j} \right)\,-\,\hat O\left( {i,j} \right)} \right]^2}}$$

Figure 7a shows the BP reconstruction calculated by Eq. (12); the original image is immersed in the noise caused by the twin image. Figure 7b, c shows the reconstructions by $\ell _1$ and TV regularization, respectively. Both methods are able to eliminate the twin image effectively, but the TV regularization result has higher contrast and smaller MSE than the $\ell _1$ regularization result. Moreover, TV regularization not only eliminates the twin image but also suppresses noise.

To evaluate the noise immunity capability, zero-mean Gaussian noise with 0.01 variance is added to the observed image. The BP reconstruction, the reconstruction by $\ell _1$ and TV regularization from a noised observed image are shown in Fig. 7d–f, respectively. The $\ell _1$ regularization result is not satisfactory due to residual noise. By contrast, TV denoising is remarkably effective in smoothing away noise while preserving edges. These results are obtained with 200 iterations. Figure 7g shows that the MSE varies with the iterations (see also Movie 1 for details). Reconstruction with TV regularization is used in our experiments.

### Fabrication

The FZA mask is fabricated on soda-lime glass substrates with a thickness of 2 mm by applying the laser direct writing technique. A Cr layer (140 nm) is deposited on the substrates by vacuum evaporation. The surface of the Cr layer is oxidized for antireflection. A photoresist layer (1 μm) is then spin-coated on the Cr layer. This blank photomask is exposed under a scanning laser beam to create a latent image in the photoresist layer. After exposure, unexposed parts of the photoresist can be removed in a developer (5–7‰ NaOH solution). Subsequently, by immersion in an etchant ((NH_4_)_2_Ce(NO_3_)_6_ + HCIO_4_ solution), the open area of the Cr layer is etched away to form a transparent zone, while the Cr layer protected by the photoresist is not etched to form an opaque zone. Finally, the remaining photoresist is stripped by immersion in a high concentration of developer.

## Supplementary information

Supplementary information (revised)

Iteration process of CS algorithm
