# Supplementary material for: Single-shot lensless imaging with fresnel zone aperture and incoherent illumination
Source: Light Sci Appl. 2020 Apr 7;9:53. doi: 10.1038/s41377-020-0289-9 (PMC7138823; doi:10.1038/s41377-020-0289-9)
Supplement: Supplementary file 1 — Supplementary information (revised) [file 41377_2020_289_MOESM1_ESM.docx]

**Single-shot Lensless Imaging with Fresnel Zone Aperture and Incoherent Illumination**

**Supplementary information**

Jiachen Wu^1^, Hua Zhang^1^, Wenhui Zhang^1^, Guofan Jin^1^,

Liangcai Cao^1,*^ and George Barbastathis^2,*^

^1^*State Key Laboratory of Precision Measurement Technology and Instruments, Department of Precision Instruments, Tsinghua University, Beijing 100084, China*

^2^*Department of Mechanical Engineering, Massachusetts Institute of Technology, 77 Massachusetts Avenue, Cambridge, Massachusetts 02139, USA*

**Corresponding author:* [*clc@tsinghua.edu.cn*](mailto:clc@tsinghua.edu.cn)*,* [*gbarb@mit.edu*](mailto:gbarb@mit.edu)

1. The derivation of Fourier expansion for FZA

Because the transmittance of FZA is only varying in radial direction, it could be treated as 1D function. Taking the quadratic term $r^{2}$ as an independent variable $x$, the transmission function of FZA could be reduced to an even square wave function whose period is $L$ and duty cycle is 50% (Fig. S1). The expression of square wave in interval $\left[-L/2,L/2\right]$ could be defined as

Fig. S1 The reduced transmission function of FZA: an even square wave function.

As an even function, it has only cosine terms in its Fourier expansion:

$$T\left(x\right)=\frac{a_{0}}{2}+\sum_{n=1}^{\infty} a_{n}\cos\left(\frac{2\pi nx}{L}\right)$$

where the Fourier coefficients are

$$a_{n}=\left\{\begin{matrix} \frac{2}{L}\int_{-\frac{L}{2}}^{\frac{L}{2}} T\left(x\right)dx, n=0 \\ \frac{2}{L}\int_{-\frac{L}{2}}^{\frac{L}{2}} T\left(x\right)\cdot\cos\left(\frac{2\pi nx}{L}\right)dx, n=1,2,3,\cdots\end{matrix}\right.$$

By substituting the Eq. into the Eq. , the Fourier coefficients could be obtained:

$$\left\{\begin{matrix} a_{0}=1 \\ a_{n}=\frac{2}{n\pi}\sin\left(\frac{n\pi}{2}\right), n=1,2,3,\cdots\end{matrix}\right.$$

Therefore, the Fourier expansion of the even square wave function is

$$\begin{matrix} T\left(x\right)=\frac{1}{2}+\frac{2}{\pi}\sum_{n=1}^{\infty} \frac{1}{n}\sin\left(\frac{n\pi}{2}\right)\cos\left(\frac{2\pi nx}{L}\right) \\ =\frac{1}{2}+\frac{2}{\pi}\cos\left(\omega x\right)-\frac{2}{3\pi}\cos\left(3\omega x\right) \\ +\frac{2}{5\pi}\cos\left(5\omega x\right)-\frac{2}{7\pi}\cos\left(7\omega x\right)+\cdots\end{matrix}$$

where $\omega=2\pi/L$. If we set $L=2r_{1}^{2}$, and $x=r^{2}$, Equation could easily be extended to 2D polar coordinate. Finally, the Fourier expansion for the transmission function of FZA could be written as

$$T\left(r\right)=\frac{1}{2}+\frac{2}{\pi}\sum_{n=1}^{\infty} \frac{1}{n}\sin\left(\frac{n\pi}{2}\right)\cos\left(\frac{\pi nr^{2}}{r_{1}^{2}}\right)$$

Figure S2 shows an approximation of an FZA pattern by taking the first 1 3, 7 and 30 terms of its Fourier series. With the increase of terms, the edges between adjacent zones become sharp. It verifies the binary FZA pattern could be decomposed into a series of Gabor zone plate (GZP) patterns, which is the reason for the multiple conjugate foci of FZA.

Fig. S2 The patterns and radial transmittance distributions of partial sums of the Fourier series for a binary transmission function of FZA. The approximations of FZA pattern take the (a) N=1, (b) N=3, (c) N=7 and (d) N=30 terms of its Fourier series, respectively.

Fig. S3 shows the simulation results about the axial intensity distribution and the cross section of the propagation of the GZP and FZA with 532 nm incident light over a range of z = 0 ~ 120 mm. Both the two zone plates shown in the middle of the figures are 2.56 × 2.56 mm, and the first order focal length is *f* = 100 mm. Though the light intensity at the focal point of GZP is less than the intensity of FZA, GZP has only one focal point (Fig. S3(a)), whereas FZA forms a higher order focal point (Fig. S3(b)). Each high-order focal position coincides well with the calculation. For FZA imaging, if propagate the hologram to high order focal plane, the silhouette of object will also be emerged. The insets in Fig. S3 show the reconstructed images at *z* = 100 mm, 33.3 mm, 20 mm, corresponding to $f$, $f/3$, $f/5$ respectively.

Fig. S3 Intensity distribution and cross section of the propagation of (a) GZP and (b) FZA with 532 nm incident light.

1. Axial resolution

Similar to compressive holography (CH), the axial resolution of our imaging system is determined by the transfer function. In CH, the transfer function under Fresnel approximation is

$$H\left(u,v\right)=\exp\left(ikz\right)\exp\left[-i\pi\lambda z\left(u^{2}+v^{2}\right)\right]$$

In this work, the transfer function is

$$H\left(u,v\right)=\exp\left[i\pi r'_{1}^{2}\left(u^{2}+v^{2}\right)\right]$$

where $r'_{1}$ is the FZA constant of the FZA shadow projected on sensor. We assume that it is approximately equal to the FZA constant *r*_1_ of the mask in the manuscript. In fact, it depends on the distance *z* from object to mask and the distance *d* from mask to sensor, that is $r'_{1}=r_{1}\left(1+d/z\right)$. Then we rewrite Eq. as

$$H_{\text{FZA}}\left(u,v;z\right)=i\exp\left[-i\pi r_{1}^{2}\left(1+\frac{d}{z}\right)^{2}\left(u^{2}+v^{2}\right)\right]$$

Let $\lambda z'=r_{1}^{2}\left(1+d/z\right)^{2}$, where $z'$ is equivalent object distance in CH. With differentiation, we could obtain

$$\lambda dz'=-2r_{1}^{2}\left(1+\frac{d}{z}\right)\frac{d}{z^{2}}dz$$

Supposing the minimum resolvable distance in CH is $\Delta z'$. According to Eq. , if $z\gg d$, the minimum resolvable distance $\Delta z$ in FZA imaging could be roughly obtained:

$$\begin{matrix} \Delta z=-\frac{\lambda z^{2}}{d}\frac{1}{2r_{1}^{2}\left(1+\frac{d}{z}\right)}\Delta z' \\ \cong-\frac{\lambda}{2r_{1}^{2}d}z^{2}\Delta z' \end{matrix}$$

It is shown that $\Delta z$ increases quadratically with the distance, which means the axial resolution decreases rapidly with the distance.

Figure S4 shows the two curves that the phase of transfer function varies with object distance, where $w=\sqrt{u^{2}+v^{2}}=1\text{mm}$, $d=3\text{mm}$, $r_{1}=0.3\text{mm}$. For FZA imaging, the phase is almost invariable and gets to the value at infinity when $z\gg d$, which has quite low axial resolution from modest distance to infinity.


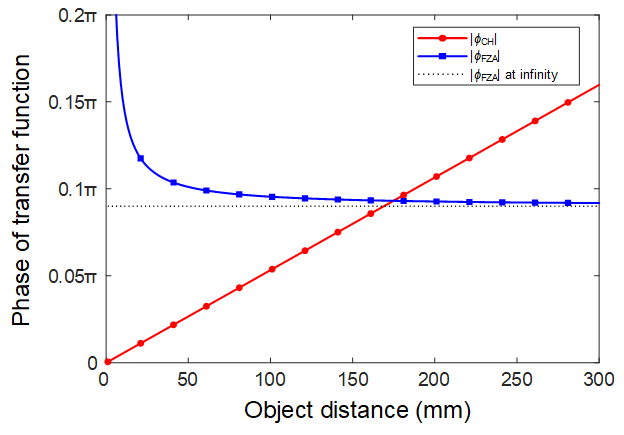


Fig. S4 Phase of transfer function vs. object distance.

Though axial resolution is low, the numerical focusing at various depths from the single-shot measurement still works. Due to the tiny frequency difference of transfer function, the reconstruction for a certain depth contains a defocused image of other depths.

1. Coherence of the illumination

The reconstructions under different wavelengths and different degrees of coherence is shown in Fig. S5. In the first row, the illumination is incoherent and the wavelength varies from 1600 nm to 100 nm. With the decrease of wavelength, the diffraction effect is weakened, and the PSNR of reconstructed images becomes higher and higher. In the second row, the coefficient of coherence varies from 1 to 0. For the high coherence illumination, the interference will produce a lot of cross terms, which results to the noise. With the decrease of the degree of coherence, the PSNR of the reconstructed images becomes higher and higher. Therefore, the illumination with low coherence and short wavelength will improve the quality of the reconstructed image. That is exactly why coded aperture methods are popular in X-ray or gamma-ray incoherent imaging.


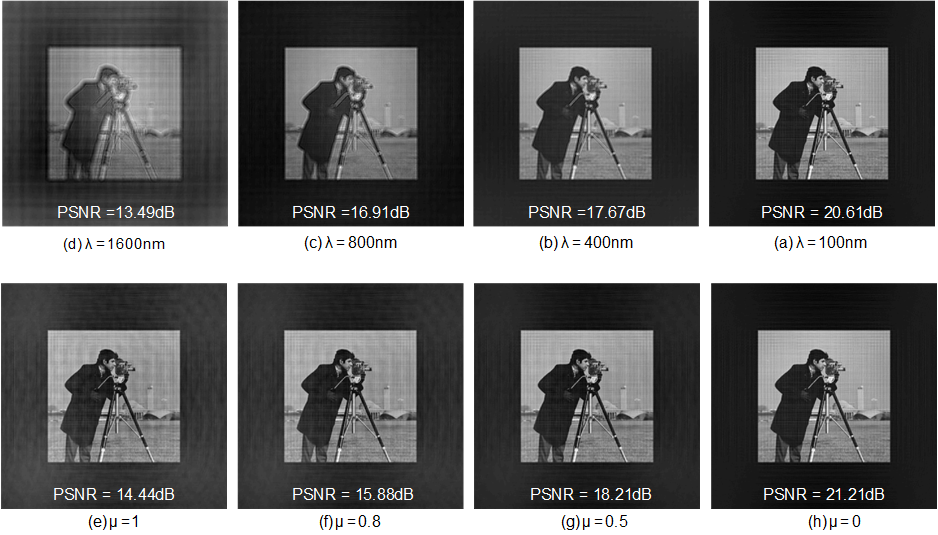


Fig. S5 The reconstructions for different wavelengths and different degrees of coherence.
